# Unplanned pregnancy at advanced maternal age: analysis in light of Transition Theory*

**DOI:** 10.1590/1980-220X-REEUSP-2024-0172en

**Published:** 2024-10-25

**Authors:** Juliane Dias Aldrighi, Hérica de Lara Cardoso, Nara Marilene Oliveira Girardon-Perlini, Herla Maria Furtado Jorge, Silvana Regina Rossi Kissula Souza, Deisi Cristine Forlin Benedett, Tatiane Herreira Trigueiro, Marilene Loewen Wall

**Affiliations:** 1 Universidade Federal do Paraná, Setor Litoral, Matinhos, PR, Brazil.; 2 Universidade Federal do Paraná, Programa de Pós-Graduação em Enfermagem, Curitiba, PR, Brazil.; 3 Universidade Federal de Santa Maria, Departamento de Enfermagem, Programa de Pós-Graduação em Enfermagem, Santa Maria, RS, Brazil.; 4 Universidade Federal do Piauí, Departamento de Enfermagem, Programa de Pós-Graduação em Enfermagem, Teresina, PI, Brazil.; 5 Universidade Federal do Paraná, Departamento de Enfermagem Materno-infantil, Programa de Pós-Graduação em Enfermagem, Curitiba, PR, Brazil.; 6 Centro Universitário Internacional, Curitiba, PR, Brazil.

**Keywords:** Maternal Age, Pregnancy, Unplanned, Nursing Theory, Pregnancy, High-Risk, Family Development Planning, Edad Materna, Embarazo No Planificado, Teoría de Enfermería, Embarazo de Alto Riesgo, Planificación Familiar

## Abstract

**Objective:**

To analyze the experience of women aged 35 or over regarding the transition to unplanned motherhood.

**Method:**

A descriptive qualitative study, with semi-structured interviews with six women in this age group, selected by convenience, carried out in a high-risk prenatal clinic between July 2022 and July 2023. Analysis was based on Transitions Theory.

**Results:**

Three main categories were identified: Transition properties (attributes of late motherhood); Transition conditions (personal, social and community factors as facilitators or inhibitors); and Response patterns (coping and adaptation strategies). The results highlight the emotional and psychosocial complexity, including the impact of social stigma, health challenges and the duality of old age, which brings both challenges and maturity.

**Conclusion:**

The need for public policies and specialized support to promote mental health, well-being and social integration is emphasized, aiming for a healthier and more empowered transition to motherhood.

## RESUMEN

**Objetivo:**

Analizar la experiencia de mujeres de 35 años o más respecto de la transición a la maternidad no planificada.

**Método:**

Estudio cualitativo descriptivo, con entrevistas semiestructuradas a seis mujeres de este grupo etario, seleccionadas por conveniencia, realizado en una clínica prenatal de alto riesgo entre julio de 2022 y julio de 2023. El análisis se basó en la Teoría de las Transiciones.

**Resultados:**

Se identificaron tres categorías principales: Propiedades de la transición (atributos de la maternidad tardía); Condiciones de transición (factores personales, sociales y comunitarios como facilitadores o inhibidores); y Patrones de respuesta (estrategias de afrontamiento y adaptación). Los resultados resaltan la complejidad emocional y psicosocial, incluido el impacto del estigma social, los desafíos de salud y la dualidad de la vejez, que trae desafíos y madurez.

**Conclusión:**

Se enfatiza la necesidad de políticas públicas y apoyo especializado para promover la salud mental, el bienestar y la integración social, apuntando a una transición a la maternidad más saludable y empoderada.

## INTRODUCTION

Women aged 35 and over face unique and complex obstacles as they experience an unexpected transition into motherhood. Social pressures, stigma, age-related pregnancy complications, balancing career and motherhood, changes in family dynamics, and reflection on priorities, expectations, and identity are just some of the difficulties these women experience^(1,2)^. Understanding these particularities is crucial to understanding the specific challenges these women face.

Unplanned pregnancy, as a public health concern, can compromise the healthy development of pregnancy. There are signs of delay in the start of prenatal care, psychological distress due to difficulty in acceptance, and an increased risk of unsafe abortions, especially when it involves women in precarious socioeconomic situations^(3,4)^. Advanced maternal age is a relevant topic in the field of nursing, as it is an event that implies clinical, emotional and social challenges for women and their families, often associated with a series of biological and obstetric conditions that increase the complexity of care and require a differentiated approach by nurses so that effective interventions are implemented, aiming at improving maternal and child outcomes and reducing associated morbidities^(5,6)^.

Thus, understanding the biopsychosocial aspects involved in transition to unplanned motherhood at an advanced maternal age is essential for nursing care, since it enables the development of woman-centered care strategies. By recognizing the feelings of ambivalence, fear, anxiety, and insecurity faced by these women, nurses can provide adequate emotional and informational support, contributing to promoting maternal and fetal health as well as implementing competent nursing care, i.e., nursing therapy^(5,7)^.

Despite the relevance of the topic, there is a gap in scientific knowledge related to the psychosocial and emotional experiences of these women as well as the adequacy and effectiveness of public policies aimed at this group. Few studies have addressed the nuances of the transitions experienced by these women^(1,8,9)^, especially in the Brazilian context^(5)^, in which specific cultural and socioeconomic factors influence such experiences. Therefore, this study seeks to mitigate these gaps, contributing to a deeper understanding and to the development of more effective and inclusive public policies that meet the needs of women in transition to late motherhood without reproductive planning in Brazil.

In this regard, it is essential to enrich knowledge about unplanned late motherhood, both nationally and internationally. This research can provide a solid theoretical and empirical basis for developing theoretical and clinical models that consider the sociocultural, psychosocial and maternal health aspects relevant to Brazilian women in this specific age group.

Thus, this study aimed to analyze the experience of women aged 35 or older regarding transition to unplanned motherhood.

## METHOD

### Study Design

This is a descriptive and qualitative study. The presentation of results was guided by the Consolidated Criteria for Reporting Qualitative Research (COREQ), recommended for research reports that use interviews or groups as a data collection technique.

This study used Afaf Meleis’ Transition Theory as a theoretical framework, which is a Middle-Range Theory in the field of nursing, which aims to understand human experiences, their reactions to changes and transitions as well as consequences on health, well-being, social role and context over time. Through a set of interconnected concepts, the theory recognizes that transitions are not isolated events, but rather complex processes that occur in several dimensions. For Meleis, transition is a passage from a relatively stable state to another^(7)^.

Transitions can be anticipated or unexpected, and can impact an individual’s physical, emotional, and psychological well-being, leading to different emotional responses and coping strategies for personal adaptations. Thus, the theory framework comprises transition types and patterns, transition properties, transition conditions (facilitating and/or inhibiting), transition response patterns (process indicators, outcome indicators), and nursing therapies^(7)^. Response patterns result from changes that occur from and through the transition, which can be positive, negative or neutral, and are related to conditions that facilitated or inhibited the transition process. Process and outcome indicators identify whether, at the end of the transition, the person feels connected, interacts while in a transition, has developed self-confidence, and has acquired mastery of new skills, identities or roles as a result of the transition. Nursing therapy is based on these attributes and translates into nursing care for individuals’ needs^(7)^. In this study, nursing therapy will not be discussed.

### Location, Population and Selection Criteria

The research was carried out at the high-risk prenatal outpatient clinic of a public university teaching hospital in southern Brazil from July 2022 to July 2023. Semi-structured interviews were conducted with six pregnant women, selected by convenience. Pregnant women (primiparous or multiparous) undergoing prenatal care at the research institution, aged 35 years or older, were included. Pregnant women not proficient in Portuguese and having planned the pregnancy were excluded.

Potential participants were identified by consulting the prenatal clinic’s schedules and checking their medical records to confirm their age. Participants were invited, upon personal request, while they were waiting for their prenatal appointment or by WhatsApp^®^ message, one day before the appointment. Thus, the research content and objective were explained and, when they showed interest in sharing their experiences, women were directed to a reserved room offered by the service. When the schedules were consulted, a total of 35 pregnant women met the inclusion criteria, of which six agreed to participate. It is worth noting that there were no exclusions, only refusals, which occurred due to pregnant women’s self-declared reason of lack of time to participate in the interview.

### Data Collection

Data were collected through a structured questionnaire with 13 sociocultural questions, such as age, sexual orientation, education, income, religion and lifestyle habits, and another 13 on gynecological and obstetric data, such as knowledge and use of contraceptive methods and family planning, pregnancy history and gestational health. Individual interviews were also conducted by telephone and three in person. The semi-structured interviews were audio-recorded and later transcribed into an editable document, and were all conducted by the first author, who has previous experience in qualitative interviews. The data collected totaled 184 minutes of recording, with an average duration of 30 minutes each.

The interview script was guided by the sentence, “Tell me how it is for you to go through the experience of being a mother at this point in your life without having planned your pregnancy”. There were nine other supporting questions that were asked if the initial response did not cover the specific topics of interest. All questions were asked to the respondents in the same order, although the interviewer conducted inductive probes based on the main responses. The script was organized into four main research areas: initial emotions and sensations; perceptions and adaptation to motherhood; support and support network; and personal development and coping.

Data collection was completed upon reaching theoretical saturation and the study objective^(10)^. The decision to conclude with six interviews was based on obtaining rich and dense data, providing a deep understanding of participants’ experiences. This number allowed us to reach theoretical saturation, in which new information would produce little or no significant revelation. Thus, the literature revealed that smaller samples, between six and 12 interviews, can be sufficient to obtain extremely accurate information with a high level of confidence, as long as there is an external truth. In the case of this research, even when dealing with opinions and perceptions, women share common experiences, and these experiences make up truths^(10)^.

### Data Analysis and Processing

Data analysis was based on Transitions Theory, and began with the complete transcription of interviews, followed by a thorough reading to be familiar with content. Inductively, i.e., allowing the data to inform the construction of categories, the researchers identified and extracted significant excerpts that reflected experiences or events representative of transitions in women’s lives. These text excerpts were grouped into preliminary categories, without imposing prior theoretical structures. Subsequently, these categories were compared and refined based on the concepts of Transitions Theory, ensuring that they were adequately aligned with theoretical principles.

The result was a set of thematic categories that not only emerged naturally from the data, but were also solidly anchored in the foundations of the underlying theory, facilitating the interpretation of participants’ transition trajectories within a coherent theoretical context. Three categories were then developed and named: Transition properties: attributes of transition to late motherhood; Transition conditions: experience of facilitating and inhibiting factors for transitioning to late motherhood; Response patterns: processes and outcomes as elements of transition to late motherhood.

### Ethical Aspects

The study was approved by the Research Ethics Committee, under Opinion 3,959,789/2020 and Certificate of Presentation for Ethical Consideration (CAAE - *Certificado de Apresentação para Apreciação* Ética) 30071720.0.0000.0096, as well as respecting all the ethical precepts set out in Resolution 466/2012 of the Brazilian National Health Council (CNS - *Conselho Nacional de Saúde*). After reading, the Informed Consent Form (ICG) was signed in two copies, attesting to participants’ expression of agreement to participate in the research.

Participants’ speeches were identified by the letter P (pregnant), followed by the number corresponding to the chronological order of the interviews, resulting in the coding P1, P2… P6, which guarantees confidentiality and anonymity in the research process.

## RESULTS

### Pregnant Woman Characterization

The mean age was 41 years, ranging from 36 to 45 years. The six women participating in this study belonged to different ethnicities (white, yellow and brown), all with heterosexual sexual orientation. Their marital status was diverse, including married, single and one single woman in a previous stable relationship. Religious affiliation was present among participants, who identified themselves as Catholic, Umbanda practitioners, spiritualists and Wiccans. Regarding education, women had levels that ranged from complete elementary school to complete higher education. The occupations were different, including housewife, cleaning assistant, administrative services, shopkeeper and civil servant, reflecting an individual income range from less than one wage to five and a half wages. All of them used the healthcare services of the Brazilian Health System (SUS – *Sistema* Único *de Saúde*), had a history of prenatal care and had a number of children that ranged from two to five, with the first pregnancy between the ages of 16 and 22. Only one was a primiparous woman in her current pregnancy. This diversity of profiles illustrates a wealth of social and economic contexts as well as previous motherhood experiences that may influence these women’s transitions and identities.

### Transition Properties: Attributes of Transition to Late Motherhood

The critical point was the moment of discovery of the unplanned pregnancy, when women became aware and recognized that their lives were undergoing a significant transformation, which triggered emotional reactions, such as crying, fear, initial shock, acceptance as well as anticipation of the challenges associated with late motherhood.

*So, everything changed, my life practically turned upside down. And I cried a lot, I cried a lot, gosh. Going through this and being a mother again at 41 years old scares me.* (P1)

*And thinking that I’m going to be a mother at 45, I also have some fears about whether I’ll have patience.* (P2)

*I didn’t expect it, I didn’t want it, but it happened, right? So, now, at the beginning, it was really hard for me. I’ll tell you that I thought about several possibilities, but my heart didn’t allow me to do any of them* [abortion]. *And now, for me, it’s okay, you know, that thing, like, for the moment, right, because, for me, it was a blow, a shock.* (P3)

*Then I start to think about how everything will change in my life after this pregnancy, and I think it will really change, you know. I always hear that when a mother is born with a baby, another person is born, so maybe another me will be born that I don’t know.* (P6)

Despite this, they showed active involvement, demonstrating engagement in the transition process, such as awareness of motherhood responsibilities and challenges, commitment to care and protection of children, and preparation and training to face the challenges of late motherhood. They demonstrated a desire for bonding, despite it being an unexpected pregnancy.

*After going through these feelings, when I think about motherhood, I think that I can’t wait to see my little princess, to hold her in my arms, to see her using everything I bought for her* […] *I want to breastfeed, you know, I really want to breastfeed her, so that’s the only information I look for.* (P3)

*I went to see a nutritionist, and prenatal care was very good, right? Because I had a slightly later pregnancy, I also went to the doctors more often. I never missed a single appointment at the prenatal clinic* […] *and then there was the high-risk prenatal care, which I did very well. I never missed any appointments or exams, precisely because of my age, which is a concern.* (P5)

*I’m doing everything right, I follow up with the doctor, I go to the health center too. Oh, and when I have time, I try to read about motherhood and parental relationships. I follow some people on Instagram* ^®^ *who talk about this, about positive parenting, about education based on nonviolent communication. I read about a lot of things because I’m concerned about the relationship I’m going to have. I don’t have children around me, I don’t work with children, so I don’t really know how to deal with children, so I think I need to see this kind of thing to start identifying with motherhood, with the responsibility of raising and educating a human being.* (P6)

By becoming aware of a transition process, women began to change their outlook on life and family dynamics, strengthening ties and improving communication. Furthermore, there were changes in their routine, which had consequences for their notions of life priorities and career/work plans. This change was seen by participants as an opportunity for learning, growth and personal transformation.

*Because it has brought the family closer together, who used to only meet at the wake, and nowadays they don’t. They send messages every day and, when they see that I haven’t posted anything, they call to ask if I’m okay.* (P1)

*I was going to start my nursing course when I found out, so I said, “Wow, that was a bombshell.”* (P3)

*My daughter is 23 years old, and I never wanted to have children again. Then I was 46 without anything. Suddenly, everything changed, and then a child came along. It’s strange, right? Everything will change. Wow, it’s already changed, but it will change, like, radically. It will change everything, everything, everything. I had nothing. I only had my job, and having to deal with that was the only thing I had, going out and traveling and that’s it. Now it’s not like that anymore. Now I have a child, I have to take care of a child, I have to raise a child, I have to start all over again. And then starting all over again at 46, as they insist on reminding me, right? At 46, everything is a big change, a big change.* (P4)

*I’m already starting to think about how everything will change in my life after this pregnancy, and I think it really will change, you know? I always hear that when a mother and baby are born, another person is born, so maybe another me will be born that I don’t know, but who will be able to handle this change. It’s my routine that will change, my lifestyle, because I won’t be able to do anything without thinking about my son. I won’t have the freedom to go out, travel and do what I want anymore; I’ll always have to plan differently.* (P6)

### Transition Conditions: Experience of Facilitating and Inhibiting Factors for Transitioning to Late Motherhood

Transitioning to late motherhood without planning was conditioned by factors that could facilitate this transition, bringing personal, community and/or social resources that served as strength to deal with the situation. Beliefs, religiosity and faith were personal tools for the positive meaning and acceptance of pregnancy.

*God has a purpose, right? At this point in the game, at my age, in my head, I say that God has a very big purpose in my life and in the life of this child, that blessings will come, and great ones.* (P1)

*To strengthen myself, I also have my religion, I am an Umbanda follower.* […] *Umbanda is my faith. In fact, it is my faith that guides me, right? So, when I feel lost, that is where I go to look for strength.* […] *we need to hold on, otherwise things won’t move forward, right?* (P3)

*I don’t have a fixed religion, like that. I like to pray, to talk to God in my own way* […] *so, that’s why I try to understand this surprise pregnancy as an opportunity to be a better person.* (P6)

Other personal factors could inhibit a successful transition, such as women’s own age, physical limitations and fatigue, failure to seek information, health problems, anxiety, fear of a cesarean section and fear of breastfeeding.

*It’s not easy, no, and age doesn’t help, right? It’s very tiring. When we’re younger, we have more energy, now it’s very tiring, and I still have asthma to make it worse.* (P1)

*This pregnancy is very different in terms of my body, pain. To walk, I have to be slower, I think it’s because of my age, it affected me a lot.* (P2)

*Physically, my body is different and, as I’m not a girl anymore, I feel the shock, I get tired, even though I’m in shape, because I’ve always exercised.* (P6)

*I didn’t even try to find out anything, because the more you find out, the more confused you become, so I didn’t look for anything, any kind of information about high-risk pregnancies, none of that, I didn’t look for anything, I abstained from all that.* (P4)

*I actually prepared myself throughout my pregnancy for a natural birth, and now I have to have a cesarean section, you know, so you prepare for something and then, in the last 45 minutes, you have to find out that you’re going to have a cesarean section, it really gets you a little nervous, but I try not to think about it.* (P5)

*I’m a little scared of breastfeeding, actually. I see some things, women saying that it’s difficult, that it requires a lot of persistence, that it hurts, that kind of thing.* (P6)

As community factors that could facilitate transition, support from family and partner as well as from the community/neighborhood and healthcare services were identified.

*The family is close-knit. Whenever needed, they help, my sisters, my son.* (P2)

*Then, in addition to the psychologist, I was dealing with family support. It was the only support I had, family, from my daughter, which was what helped, but nothing more than that.* (P4)

*I’m getting help, right? My mother is helping, my mother is here with me, and my husband too, right, he took a vacation now to be able to help his mother.* (P5)

*Sometimes my legs lock up and I can’t walk, and I have to lie down and I tell them to call for help, a neighbor, or the people at the health center. The people gave me the health center’s WhatsApp number, so I send a message saying if I’m not well, and then the doctor comes to see me* […] *the neighbors are attentive, so I can even count on them for any emergency.* (P1)

*I was quite scared, then they* [doctor and nurse] *talked to me a lot and I am calmer now.* (P2)

Some community factors could inhibit the transition, such as being a single mother and lack of family/partner support.

*Then when he* [the baby’s father] *found out I was pregnant, he came to my house. We tried to stay together, but the last ultrasound showed it was a girl, and since he always wanted a boy, I don’t know if he was sad, or what he thought in his head, so now he doesn’t even answer the phone.* […] *when you have a mother, you cling to your mother, your mother is your support. But I was left alone, I have no one to talk to, no one to lean on. Sometimes I’m in pain and I have to stay quiet, I have to put up with it.* (P1)

*It was difficult, because, well, it was a relationship in which I wasn’t married to him, right, we were just dating and men just jump ship, right, that’s basic. So, since I’m already 36 years old, I already know that. If a married man jumps ship, imagine what’s not like, right?* (P3)

In addition to other factors, social dimensions can interfere with inhibiting transition. Some women lived in precarious socioeconomic conditions, with financial difficulties. In addition, they often faced social barriers, such as the embarrassment of becoming pregnant at an older age and stigma associated with the idea of being an “older mother”. There was also an unfair blame placed on them for conceiving at this stage of life. These issues were aggravated by social and cultural impositions surrounding motherhood, often seen as a socially expected role for women.

*The other day he called her and I took advantage and said that I needed to buy the baby’s things, diapers and everything, because I don’t have anything, so he sent 500 reais. But he thinks that these 500 reais have to last a lifetime.* (P1)

*It’s a question they ask, but everyone asks it. Wow, how do you get pregnant at 46? But how are you going to handle it now?* […] *I’m going to be 47 next year, at the beginning of next year, that’s what worries me. My daughter-in-law is also pregnant, so sometimes I feel embarrassed, I feel too old to be pregnant. I wasn’t expecting it, right?* (P2)

It should be noted that facilitating conditions at a social level were not identified in speeches.

### Response Patterns: Processes and Outcomes as Elements of Transition to Late Motherhood

The feeling of connection and interaction was revealed through faith and religiosity, relationships with family, community/neighbors, in order to seek support in the transition process, in addition to relationships with healthcare professionals, demonstrating engagement with healthcare and the search for information to connect with transition.

*I talk to God a lot. Just yesterday, I was lying down and thinking to God, “How can a human being be inside us?”* […] *will I be able to do it, will I win? I talk to God a lot. He has been my daily companion.* (P1)

*I went with myself trying to understand, accept, reflect on life, on the things that happen. I didn’t seek any psychological help, only my family.* (P6)

*So, my family is not a support point, it is more the people in the neighborhood who help me, more than my family, if I need it.* (P1)

*The doctor and nurse at the clinic who are monitoring my pregnancy give me guidance and talk to me. I was scared, and they calmed me down. I feel comfortable with them, they make me feel very safe.* (P2)

*I’m getting information, and I also get a lot of questions answered during my appointments. The doctor is very direct. The nurse is a little more detailed. She explains a few things in more detail.* (P6)

Through these connections and interactions, women developed self-confidence and coping strategies to transition to unplanned late motherhood, such as the ability to deal with the challenges of late pregnancy, even for single mothers. They demonstrated personal maturity and understood the entire process as a learning experience for life.

*I already knew everything I was going to go through alone, everything I’m going to go through in fact, and that was my despair* […]*, but today I see myself as completely free, I’m completely independent* […] *I believe I’ve matured a lot now with this pregnancy. It helped me finish this maturation, you know?* (P3)

*I only have this alternative, it’s to be a mother or to be a mother, there is no other, so I’m confident, I have to be, right? We make mistakes, there’s no way around it, we made mistakes in the first one, we’ll make mistakes in the second one, but I’m very confident* […], *at least try to do something good, a good job, a good education.* (P4)

*I still don’t know exactly what the learning will be, but for now, this is what it is, even if we plan, have a path in mind, things can change and we need to adapt, open ourselves up to a new experience that wasn’t in our plans, but that can be very good.* (P6)

Thus, there was also the development of the capacity to adapt to new challenges, roles, behaviors and needs imposed by transition, translated into a certain mastery of the situation. This also demonstrated women’s capacity to experience, function and be well in other areas of life, despite the transitional condition and all its ambiguities, as a result of a fluid and integrative identity.

*So, I’m really lost, but I’m managing, I’m slowly learning how to be a mother, it’s something new, I’m learning everything, it’s like my first pregnancy.* (P1)

*I said, “No, I can’t, I’ll do it alone, I’ll fight alone, I can”* […] *we develop strength to face things, I think we always have to develop, because otherwise we won’t move forward, right? I had to develop, I had to adapt to what was happening to me.* (P3)

*It’s best to try to see things as a process that is being built. We understand, adapt and try to make it work in the way that fits into our lives, our way of living.* […] *I try to think about this pregnancy, like this, with my feet on the ground and a clear head, calm down and get it into my head that it was meant to be this way and face it in the best way.* (P6)

*I have my income, my job, I study, I work as a nursing technician, I take care of my family, so I don’t depend on anyone, me and my children.* (P3)

## DISCUSSION

Transition to unplanned late motherhood involves emotional and psychosocial complexities. The discourses show that the discovery of an unexpected pregnancy was the entry point or critical event that triggered awareness of transition to late motherhood. At this time, women realize the significant change in their lives. The lack of planning of these pregnancies, both due to failures in the health system and the lack of adherence of women and sexual partners to health actions, has an impact on the lives of women and families^(11)^, and can interfere with transition of this moment that could be experienced calmly. The issue of age refers to anomalies, syndromes and the inability to give birth naturally. Daily life also causes concern, as it alters relationships, finances and work^(5,12)^.

Uncertainties, emotional anguish and interpersonal conflicts, reported by participants, are markers of this transitional process, as profound changes are moving individuals’ life experiences so that a new reality can develop^(7)^. It is understood that the transitions that occur in the lives of these women are of different types and patterns and are interconnected, such as work, relationship and age transitions, which are characterized as situational, with a general change in life, relationships, as well as career, and developmental transitions, as they need to adapt to new roles and reconfigure their identities, as well as health/illness transitions, when they report physical impact, health challenges and emotional concerns.

Thus, transitions also occurred in multiple patterns, both sequential and simultaneous, influencing each woman’s experience. Most pregnant women face multiple transitions, as they are not only adjusting to the idea of being a mother again, often after a long interval, but are also dealing with situational, developmental, and health/illness changes. Thus, pregnant women’s transition experiences are clearly interconnected and begin with the discovery of pregnancy, which leads to personal and emotional adjustments, followed by physical and social adaptations. This progression reflects the sequential and simultaneous transition of women through several stages of adaptation. Thus, nursing plays an important role in supporting this period of transition by sharing information and providing specific nursing care that is linked to each woman’s individual needs.

Transition to motherhood in older pregnancies, especially unplanned ones, can be facilitated by personal conditions. Faith and religiosity have been shown to be a spiritual dimension that can facilitate processes, resulting in favorable psychological effects, thanks to belief, which in turn helps to increase self-confidence and acceptance of the challenge imposed and, thus, have a positive impact on the transition process, especially on mental health^(12)^.

However, personal factors can also make this transition difficult, as exemplified in speeches, such as the pressure of age, physical limitations linked to this age, fatigue, and pre-existing illnesses. Some women who become pregnant at an advanced age may experience this type of situation, especially if they did not plan their pregnancy. They may question the failure of contraception, which is a socially assigned responsibility of women, and that, by becoming pregnant at the “wrong” age, they put the baby’s health at risk^(5,12)^.

Furthermore, when it comes to late pregnancy, women may feel embarrassed and stigmatized for being “old” mothers^(6)^. The Ministry of Health recommends that the “ideal” age for becoming pregnant is between 20 and 34 years old, since after that, there is a risk of maternal-fetal complications^(13)^. However, age alone may not be a risk factor if a woman is healthy and has good prenatal care^(5)^. Furthermore, there is a sociocultural construction that older women or women close to the menopause period do not have a sexual life or are unable to become pregnant due to decreased fertility^(8)^.

Late pregnancy, despite being a trend, still causes astonishment, and women in this situation are the target of insensitive and prejudiced comments^(6,8)^. Such situations can result in disconnection from motherhood and isolation, preventing the development of adaptive skills, indicative of a challenging transition.

Community conditions, such as social support from family and partners, play an essential role as socializing agents in people’s lives, transmitting not only support and guidance, but also values, beliefs and traditions. This interrelationship and interaction with micro and macro systems helps or hinders the transition process, as do other social contexts that include extended networks, such as neighbors, friends and healthcare services^(12,14,15)^.

In Brazil, there is a considerable proportion of women who are mothers but are not involved in a formal marriage or conjugal relationship. This can occur both due to intentional choices and for sociocultural reasons, such as abandonment or lack of involvement of fathers in raising their children. Single motherhood is a social problem and a significant reality in Brazil, where they often face a series of unequal and discriminatory experiences that affect them. This includes stigma associated with lack of a partner, along with other factors that have a considerable impact on their daily lives, such as obstacles to accessing certain social instances, a heavier workload and salary disparities^(16)^.

All of these factors, coupled with age stigma and the lack of other types of social support, can trigger process and outcome indicators that lead women to vulnerabilities, especially psychosocial ones, preventing them from developing self-confidence, coping strategies, and situational control. These process and outcome indicators are essential for assessing women’s ability to manage the changes arising from transition, which require new skills, feelings, goals, behaviors, or functions^(7)^.

Hence, it was clear that the unexpected pregnancy triggered a profound review of their self-image and social roles. This identity flux, which is based on transformation and self-perception, is observed in the discourses, in which they articulate feelings of uncertainty and redefinition. Transition is not only physical, but deeply rooted in psychological and social dimensions, influenced both by internal factors and by the support (or lack thereof) of the social environment. The interviewees reflect on how late motherhood changed their priorities, life perspectives and interpersonal relationships, illustrating how female identity, particularly in contexts of unplanned motherhood, is in constant evolution and renegotiation.

Recent years have seen a significant shift in perceptions of late-life pregnancy and women’s roles in society. Historically, women were expected to have children at a younger age, but the increase in the mean age of first pregnancy reflects changes in women’s priorities and educational and career opportunities. These changes also pose unique mental health challenges, such as stress associated with balancing career and motherhood, and the impact of social stigma on women who choose to have children at an older age^(9)^.

Literature suggests that women in late pregnancy may experience heightened levels of anxiety and stress, often exacerbated by the perception that they are “out of time” for motherhood. In addition, these women may feel additional pressure to meet social expectations of being successful mothers and professionals, which can negatively impact their mental health^(14)^. It is therefore crucial that adequate psychological and social support is provided, including the implementation of public policies that recognize and address these specific needs. Psychological support programs, support groups, and specialized counseling can be important tools to help these women overcome the emotional and social challenges of late pregnancy.

Recent research has highlighted the importance of recognizing and addressing the psychosocial and emotional aspects of late-life motherhood. Studies suggest that targeted interventions can not only improve pregnant women’s mental health, but also promote a more positive and satisfying motherhood experience^(12,14)^.

It is essential that healthcare professionals, especially nurses, are aware of these complexities of transition to unplanned late motherhood and provide nursing therapies to facilitate and inspire healthy processes and positive transition responses. This includes nursing interventions based on health education, accurate information and guidance, empowering women to cope, setting goals, emotional support and, when necessary, referral to psychological support services or social assistance. For this to occur, there must be interaction and dialogue between nurses and women/families in order to identify milestones and critical points, support networks and other individualized needs.

The study contributes to the advancement of nursing knowledge as it highlights the psychosocial and emotional complexity of unplanned late motherhood, highlighting the multiple dimensions that affect women during this transition. Therefore, it is essential that nursing broadens its perspective beyond the physiological and pathological aspects of pregnancy, addressing the subjective, behavioral and interpersonal dimensions. Identifying specific areas for support and nursing interventions guided by the perspective of self-care, quality of life, well-being and skill development is crucial to prepare women for issues that transcend biomedical concerns.

Furthermore, reflecting on the current reproductive planning policy in Brazil, we can see the need for a more integrated and fragmented approach. The provision of contraceptives, although important, is only one facet of a more comprehensive public policy that must include educational, guidance and clinical actions, forming an indivisible tripod for sexual and reproductive health protection. Therefore, it is urgent to strengthen public policies in this area, with government incentives and modernization of operationalization strategies by the Brazilian Healthcare Network.

Since transition occurs over time, it is understood that a limitation may have been data collection only during pregnancy, which may have impaired the perception of the experience of these women after childbirth, when, inferentially, transition may be completed. Moreover, data from only one region and in small quantities may limit generalization. However, it is understood that the findings presented here cannot be disregarded, as they present important experiences about the gestational period of a population that has been little studied in Brazil.

Furthermore, it is worth highlighting as a limitation the difficulty pregnant women have in accepting to carry out interviews, even if the telephone option were offered, in order to allow each woman flexibility in terms of time.

## CONCLUSION

This study deepens our understanding of the challenges faced by women undergoing an unplanned transition to motherhood at an older age by providing insights into their experiences, highlighting the complexity of the adaptation process, based on a variety of factors that can facilitate or inhibit a successful transition to late motherhood. This will help to reflect on individualized interventions aimed at supporting women through specific guidance on antenatal care, family planning and coping strategies.

Future research can further explore the nuances of this experience, aiming to improve care practices and contribute to the development of more effective public policies. Brazilian nursing can advance in the development of specialized training, development of care protocols that consider the particularities of late pregnancy and foster continued research in the area on best practices. Finally, this study highlights the importance of approaching unplanned late motherhood with awareness and understanding, considering the unique needs of these women and their families, in search of a healthier and more empowering transition process.
