# Supplementary material for: Unplanned pregnancy at advanced maternal age: analysis in light of Transition Theory
Source: Rev Esc Enferm USP. 2024 Oct 25;58:e20240172. doi: 10.1590/1980-220X-REEUSP-2024-0172en (PMC11524157; doi:10.1590/1980-220X-REEUSP-2024-0172en)
Supplement: Translation

# Translation: Gravidez não planejada em idade materna avançada: análise à luz da Teoria das Transições*

**DOI:** 10.1590/1980-220X-REEUSP-2024-0172pt

**Authors:** Juliane Dias Aldrighi, Hérica de Lara Cardoso, Nara Marilene Oliveira Girardon-Perlini, Herla Maria Furtado Jorge, Silvana Regina Rossi Kissula Souza, Deisi Cristine Forlin Benedett, Tatiane Herreira Trigueiro, Marilene Loewen Wall

**Affiliations:** 1 Universidade Federal do Paraná, Setor Litoral, Matinhos, PR, Brasil.; 2 Universidade Federal do Paraná, Programa de Pós-Graduação em Enfermagem, Curitiba, PR, Brasil.; 3 Universidade Federal de Santa Maria, Departamento de Enfermagem, Programa de Pós-Graduação em Enfermagem, Santa Maria, RS, Brasil.; 4 Universidade Federal do Piauí, Departamento de Enfermagem, Programa de Pós-Graduação em Enfermagem, Teresina, PI, Brasil.; 5 Universidade Federal do Paraná, Departamento de Enfermagem Materno-infantil, Programa de Pós-Graduação em Enfermagem, Curitiba, PR, Brasil.; 6 Centro Universitário Internacional, Curitiba, PR, Brasil.

**Keywords:** Idade Materna, Gravidez não Planejada, Teoria de Enfermagem, Gravidez de Alto Risco, Planejamento Familiar

## Abstract

**Objetivo:**

Analisar a experiência de mulheres com 35 anos ou mais acerca da transição para a maternidade não planejada.

**Método:**

Estudo qualitativo descritivo, com entrevistas semiestruturadas com seis mulheres nessa faixa etária, selecionadas por conveniência, realizado em um ambulatório de pré-natal de alto risco entre julho de 2022 e julho de 2023. A análise foi baseada na Teoria das Transições.

**Resultados:**

Identificaram-se três categorias principais: Propriedades da transição (atributos da maternidade tardia); Condições da transição (fatores pessoais, sociais e comunitários como facilitadores ou inibidores); e Padrões de resposta (estratégias de enfrentamento e adaptação). Os resultados destacam a complexidade emocional e psicossocial, incluindo impacto do estigma social, desafios de saúde e a dualidade da idade avançada, que traz desafios e maturidade.

**Conclusão:**

Enfatiza-se a necessidade de políticas públicas e suporte especializado para promover saúde mental, bem-estar e integração social, visando uma transição mais saudável e empoderada para a maternidade.

## INTRODUÇÃO

Mulheres com 35 anos ou mais enfrentam obstáculos únicos e complexos ao vivenciar uma transição inesperada para a maternidade. As pressões sociais, o estigma, as complicações na gestação advindas da idade, a conciliação entre carreira e maternidade, as mudanças na dinâmica familiar, a reflexão sobre prioridades, as expectativas e a identidade são algumas dificuldades vivenciados por essas mulheres^(1,2)^. Compreender essas particularidades é crucial para entender os desafios específicos dessas mulheres.

A gravidez não planejada, como um problema de saúde pública, pode comprometer o desenvolvimento saudável da gestação. Há indícios de atraso no início do pré-natal, desgaste psicológico devido à dificuldade de aceitação, aumento do risco de abortos inseguros, principalmente quando se trata de mulheres em situação socioeconômica precária^(3,4)^. A idade materna avançada é um tema relevante na área da enfermagem, pois se trata de um evento que implica desafios clínicos, emocionais e sociais para as mulheres e suas famílias, muitas vezes associado a uma série de condições biológicas e obstétricas que ampliam a complexidade do cuidado e exigem abordagem diferenciada por parte da enfermeira, para que intervenções efetivas sejam implementadas, visando a melhoria dos desfechos materno-infantis e redução das morbidades associadas^(5,6)^.

Assim, compreender aspectos biopsicossociais envolvidos na transição para a maternidade não planejada em idade materna avançada é essencial para o cuidado de enfermagem, uma vez que possibilita o desenvolvimento de estratégias de cuidado centradas na mulher. Ao reconhecer os sentimentos de ambivalência, medo, ansiedade e insegurança enfrentados por essas mulheres, a enfermeira pode fornecer suporte emocional e informacional adequado, contribuindo para a promoção da saúde materna e fetal, assim como para a realização do cuidado de enfermagem competente, ou seja, a terapêutica de enfermagem^(5,7)^.

Apesar da relevância do tema, percebe-se uma lacuna no conhecimento científico relacionado às experiências psicossociais e emocionais dessas mulheres, assim como a adequação e efetividade das políticas públicas voltadas para esse grupo. Poucos estudos têm abordado as nuances das transições vivenciadas por essas mulheres^(1,8,9)^, especialmente em contexto brasileiro^(5)^, em que fatores culturais e socioeconômicos específicos influenciam tais experiências. Portanto, este estudo busca mitigar essas lacunas, contribuindo para uma compreensão mais profunda e para o desenvolvimento de políticas públicas mais efetivas e inclusivas que atendam às necessidades das mulheres em transição para a maternidade tardia sem planejamento reprodutivo no Brasil.

Nesse sentido, é essencial enriquecer o conhecimento sobre a maternidade tardia não planejada, tanto em âmbito nacional quanto em âmbito internacional. Esta pesquisa pode subsidiar uma base teórica e empírica sólida para o desenvolvimento de modelos teóricos e clínicos que considerem os aspectos socioculturais, psicossociais e de saúde materna relevantes para as mulheres brasileiras nessa faixa etária específica.

Assim, o objetivo deste estudo foi analisar a experiência de mulheres com 35 anos ou mais acerca da transição para a maternidade não planejada.

## MÉTODO

### Desenho do Estudo

Estudo descritivo e qualitativo. A apresentação dos resultados foi orientada pelo *Consolidated Criteria for Reporting Qualitative Research* (COREQ), recomendado para relatos de pesquisa que utilizam entrevistas ou grupos como técnica de coleta de dados.

Este estudo utilizou a Teoria das Transições de Afaf Meleis como referencial teórico, que é uma Teoria de Médio Alcance no âmbito da enfermagem, a qual pretende compreender as vivências humanas, suas reações diante de mudanças e transições, bem como as consequências na saúde, bem-estar, papel social e contexto ao longo do tempo. Por meio de um conjunto de conceitos interconectados, a teoria reconhece que as transições não são eventos isolados, mas sim processos complexos que ocorrem em várias dimensões. Para Meleis, a transição é uma passagem de um estado relativamente estável para outro^(7)^.

As transições podem ser antecipadas ou inesperadas, e podem ter impacto no bem-estar físico, emocional e psicológico de um indivíduo, levando a diferentes respostas emocionais e estratégias de enfrentamento para adaptações pessoais. Assim, a estrutura da teoria compreende os tipos e padrões da transição, as propriedades da transição, as condições da transição (facilitadoras e/ou inibidoras), padrões de resposta da transição (indicadores de processo, indicadores de resultado) e terapêuticas de enfermagem^(7)^. Os padrões de resposta resultam das mudanças ocorridas a partir e por meio da transição, que podem ser positivas, negativas ou neutras, e estão relacionados às condições que facilitaram ou inibiram o processo de transição. Os indicadores de processo e de resultados identificam se, ao final da transição, a pessoa se sente conectada, interage ao estar situada em uma transição, se desenvolveu autoconfiança, se adquiriu o domínio de novas competências, identidades ou papéis em consequência da transição. A terapêutica de enfermagem está baseada nesses atributos, e se traduz em cuidado de enfermagem às necessidades dos indivíduos^(7)^. Neste estudo, não será discutida a terapêutica de enfermagem.

### Local, População e Critérios de Seleção

A pesquisa foi realizada no ambulatório de pré-natal de alto risco de hospital-escola de universidade pública do sul do Brasil no período de julho de 2022 a julho de 2023. Foram realizadas entrevistas semiestruturadas com seis gestantes, selecionadas por conveniência. Os critérios de inclusão foram gestantes (primíparas ou multíparas) em acompanhamento pré-natal na instituição pesquisada, com idade de 35 anos ou mais. Os critérios de exclusão foram não dominar o idioma português e ter planejado a gestação.

Potenciais participantes foram identificadas por meio da consulta às agendas do ambulatório de pré-natal e verificação dos prontuários para confirmação da idade. As participantes foram convidadas, mediante solicitação pessoal, enquanto aguardavam atendimento para a consulta de pré-natal ou por mensagem de *WhatsApp* ^®^, um dia antes da consulta. Assim, foram explicados o conteúdo e o objetivo da pesquisa e, ao mostrarem interesse em compartilhar suas experiências, as mulheres foram direcionadas a uma sala reservada ofertada pelo serviço. Nos momentos em que as agendas foram consultadas, um total de 35 gestantes atenderam aos critérios de inclusão, das quais seis aceitaram participar. Ressalta-se que não houve exclusões, apenas recusas, que aconteceram por motivo autodeclarado das gestantes de falta de tempo para participar da entrevista.

### Coleta de Dados

Os dados foram coletados por meio de um questionário estruturado, com 13 perguntas socioculturais, como idade, orientação sexual, escolaridade, renda, religião e hábitos de vida, e outras 13 sobre dados ginecológicos e obstétricos, como conhecimento e uso de métodos contraceptivos e planejamento familiar, histórico de gestações, e saúde gestacional. Também foram realizadas entrevistas individuais por ligação telefônica e três de forma presencial. As entrevistas semiestruturadas foram audiogravadas e, posteriormente, transcritas em documento editável, e foram todas realizadas pela primeira autora, que possui experiência prévia em entrevistas qualitativas. Os dados coletados totalizaram 184 minutos de gravação, com duração média de 30 minutos cada.

O roteiro de entrevista foi norteado pela sentença: “Fale-me como está sendo para você passar pela experiência de ser mãe neste momento da vida sem ter planejado sua gestação”. Havia outras nove perguntas de suporte que eram aplicadas caso a resposta inicial não contemplasse os temas específicos de interesse. Todas as perguntas foram feitas às respondentes na mesma ordem, embora a entrevistadora realizasse sondagens indutivas baseadas nas respostas principais. O roteiro estava organizado em quatro áreas principais de pesquisa: emoções e sensações iniciais; percepções e adaptação à maternidade; suporte e rede de apoio; e desenvolvimento pessoal e enfrentamento.

A coleta de dados foi concluída ao alcançar a saturação teórica e o objetivo do estudo^(10)^. A escolha de concluir com seis entrevistas foi baseada na obtenção de dados ricos e densos, proporcionando uma compreensão profunda das experiências das participantes. Este número permitiu alcançar a saturação teórica, em que novas informações produziriam pouca ou nenhuma revelação significativa. Nesse sentido, a literatura revela que amostras menores, entre seis e 12 entrevistas, podem ser suficientes para alcançar informações extremamente precisas com um alto nível de confiança, contanto que haja uma verdade externa. No caso desta pesquisa, mesmo tratando-se de opiniões e percepções, as mulheres compartilham experiências comuns, e essas experiências compõem verdades^(10)^.

### Análise e Tratamento dos Dados

A análise dos dados ocorreu com base na Teoria das Transições, e iniciou-se com a transcrição completa das entrevistas, seguida de leitura minuciosa para familiarização com o conteúdo. De maneira indutiva, isto é, permitindo que os dados informassem a construção das categorias, as pesquisadoras identificaram e extraíram trechos significativos que refletiam experiências ou eventos representativos de transições na vida das mulheres. Esses extratos de texto foram agrupados em categorias preliminares, sem imposição de estruturas teóricas prévias. Posteriormente, essas categorias foram comparadas e refinadas com base nos conceitos da Teoria das Transições, garantindo que se alinhassem adequadamente aos princípios teóricos.

O resultado foi um conjunto de categorias temáticas que não só emergiram naturalmente dos dados, mas também estavam solidamente ancoradas nos fundamentos da teoria de base, facilitando a interpretação das trajetórias de transição das participantes em um contexto teórico coerente. Então, três categorias foram desenvolvidas e denominadas: Propriedades da transição: atributos da transição à maternidade tardia; Condições da transição: vivência de fatores facilitadores e inibidores para transicionar à maternidade tardia; Padrões de resposta: processos e resultados como elementos de travessia para a transição à maternidade tardia.

### Aspectos Éticos

O estudo foi aprovado pelo Comitê de Ética em Pesquisa, sob Parecer n.º 3.959.789/2020 e Certificado de Apresentação para Apreciação Ética (CAAE) n.º 30071720.0.0000.0096, bem como respeitou todos os preceitos éticos dispostos na Resolução n.º 466/2012 do Conselho Nacional de Saúde (CNS). Após a leitura, o Termo de Consentimento Livre e Esclarecido (TCLE) foi assinado em duas vias, atestando a manifestação pela concordância da participante em compor a pesquisa.

Os discursos das participantes foram identificados pela letra G (gestante), seguida do numeral correspondente à ordem cronológica das entrevistas, resultando na codificação G1, G2…G6, o que garante o sigilo e o anonimato no processo de pesquisa.

## RESULTADOS

### Caracterização das Gestantes

A média de idade foi de 41 anos, variando de 36 a 45 anos. As seis mulheres participantes desta pesquisa pertenciam a diferentes etnias (branca, amarela e parda), todas com orientação sexual heterossexual. A situação conjugal foi diversa, incluindo casadas, solteiras e uma solteira com união estável anterior. A religiosidade estava presente entre as participantes, que se identificaram como católicas, umbandistas, espíritas e wiccanista. No que se refere à educação, as mulheres apresentaram níveis que variaram do ensino fundamental completo ao ensino superior completo. As ocupações foram distintas, abrangendo dona de casa, auxiliar de limpeza, serviços administrativos, comerciante e servidora pública, refletindo uma variação de renda individual de menos de um salário a cinco salários e meio. Todas faziam uso dos serviços de saúde do Sistema Único de Saúde (SUS), com histórico de pré-natal e uma quantidade de filhos que variou de dois a cinco, com a primeira gravidez entre os 16 e 22 anos. Apenas uma era primípara na gestação atual. Essa diversidade de perfis ilustra uma riqueza de contextos sociais e econômicos, bem como experiências de maternidade anteriores que podem influenciar as transições e as identidades dessas mulheres.

### Propriedades da Transição: Atributos da Transição à Maternidade Tardia

O ponto crítico foi o momento da descoberta da gestação não planejada, em que as mulheres apresentaram consciência e reconheceram que suas vidas estavam passando por uma transformação significativa, o que desencadeou reações emocionais, como choro, medo, choque inicial, aceitação, bem como antecipação dos desafios associados à maternidade tardia.

*Então, mudou tudo, praticamente a minha vida virou de pernas para o ar. E eu chorei bastante, chorei muito, nossa. Passar por isso de ser mãe de novo aos 41 anos me deixa assustada*. (G1)

*E pensando que eu vou ser mãe aos 45 anos, passa uns medos na cabeça também, se vou ter paciência.* (G2)

*Não esperava, não queria, porém aconteceu, né, então, agora, assim, no comecinho, foi bem difícil para mim, vou te dizer que eu pensei em várias possibilidades, mas meu coração não me permitiu nenhuma delas* [aborto]. *E agora, para mim, tá tranquilo, sabe, aquela coisa, assim, de momento, né, porque, para mim, foi um baque, um choque.* (G3)

*Aí já começo a pensar como tudo vai mudar na minha vida depois dessa gravidez, e acho que vai mudar de verdade, sabe. Eu ouço sempre que nasce uma mãe com o bebê, nasce uma outra pessoa, então talvez nasça outra de mim que eu não conheço.* (G6)

Apesar disso, apresentaram um envolvimento ativo, demonstrando engajamento no processo transicional, como consciência sobre as responsabilidades e desafios da maternidade, comprometimento no cuidado, proteção dos filhos, em se preparar e se capacitar para enfrentar os desafios da maternidade tardia. Demonstraram desejo de vínculo, apesar de ser uma gestação inesperada.

*Depois de passar por esses sentimentos, quando eu penso na maternidade, eu penso que não vejo a hora de ver minha princesinha, de pegar ela no colo, de ver ela usando tudo que eu comprei para ela* […] *eu quero amamentar, sabe, eu quero muito amamentar ela, então é a única informação que eu procuro.* (G3)

*Fui atrás de nutricionista, o pré-natal foi muito bem-feito, né. Por ter tido uma gestação um pouco mais tarde, eu também fui mais vezes nos médicos. No pré-natal do postinho, nunca faltei em nenhuma consulta* […] *e aí teve o pré-natal de alto risco também que eu fiz certinho, não faltava às consultas, aos exames, justamente por conta da idade que preocupa.* (G5)

*Tô fazendo tudo certinho, acompanho com a médica, vou no posto também. Ah, eu procuro ler quando tenho tempo sobre maternidade, sobre relações parentais. Eu sigo umas pessoas no Instagram* ^®^ *que falam sobre isso, sobre parentalidade positiva, sobre educação a partir da comunicação não violenta. Eu leio sobre várias coisas, porque me preocupo com essa relação que eu vou ter. Eu não tenho crianças ao meu redor, não trabalho com crianças, então não sei exatamente lidar com criança, aí acho que preciso ver esse tipo de coisa pra começar a me identificar com a maternidade, com a responsabilidade de criar e educar um ser humano.* (G6)

Ao tomar consciência de um processo de transição, as mulheres iniciaram uma mudança nas perspectivas de vida, na dinâmica familiar, com estreitamento de laços e melhoria na comunicação. Além disso, houve as mudanças na rotina, que tiveram consequências nas noções de prioridades de vida e planos de carreira/trabalho. Essa mudança foi vista pelas participantes como uma oportunidade de aprendizado, crescimento e transformação pessoal.

*Porque já uniu mais a família, que só se encontrava em velório, e hoje em dia não, eles mandam mensagens todos os dias e, quando eles veem que eu não postei nada, eles ligam perguntando se eu estou bem.* (G1)

*Eu ia começar meu curso de enfermagem quando eu descobri, então eu falei: “Nossa, foi uma bomba”.* (G3)

*Minha filha tem 23 anos, e eu nunca mais quis ter filho, aí eu fui até os 46 sem nada; de repente, mudança de tudo, aí vem uma criança, é estranho, né? Vai mudar tudo, nossa, já mudou, mas vai mudar, assim, radicalmente, vai mudar tudo, tudo, tudo. Eu não tinha nada, eu só tinha meu trabalho, e ter que lidar com ali só era a única coisa que tinha, passear e viajar e só, agora não é mais assim, agora tem uma criança, tem que cuidar de uma criança, tem que educar uma criança, tem que começar tudo de novo, aí começar tudo de novo com 46 anos, como insistem em lembrar, né? Com 46 anos, as coisas todas é uma mudança muito, uma mudança grande.* (G4)

*Aí já começo a pensar como tudo vai mudar na minha vida depois dessa gravidez, e acho que vai mudar de verdade, sabe? Eu ouço sempre que nasce uma mãe com o bebê, nasce uma outra pessoa, então talvez nasça outra de mim que eu não conheço, mas que vai dar conta dessa mudança. É a rotina que vai mudar, é o estilo de vida, porque não vou poder fazer nada sem pensar no meu filho. Eu não vou mais ter a liberdade de sair, viajar e fazer o que eu quero; vou sempre ter que planejar de outra forma.* (G6)

### Condições da Transição: Vivência de Fatores Facilitadores e Inibidores Para Transicionar à Maternidade Tardia

Transicionar para a maternidade tardia sem planejamento estava condicionado a fatores que poderiam facilitar essa transição, trazendo recursos de âmbito pessoal, comunitário e/ou social que servissem como fortalecimento para lidar com a situação. As crenças, a religiosidade e a fé foram ferramentas pessoais para a significação positiva e aceitação da gestação.

*Deus tem um propósito, né? Nessas alturas do campeonato, na idade que eu estou, na minha cabeça, eu digo que Deus tem um propósito muito grande na minha vida e na vida dessa criança, que vão vir bênçãos e das grandes.* (G1)

*Pra me fortalecer, também tenho a minha religião, sou umbandista.* […] *a Umbanda é a minha fé. Na verdade, é a minha fé que me guia, né? Então, quando eu me sinto perdida, é onde eu vou procurar a força.* […] *a gente precisa se apegar, senão as coisas não caminham, né?* (G3)

*Eu não tenho uma religião fixa, assim. Eu gosto de orar, de falar com Deus da minha forma* […] *então, por isso que eu tento entender essa gravidez surpresa como uma oportunidade de ser uma pessoa melhor.* (G6)

Outros fatores pessoais poderiam *inibir* uma transição bem-sucedida, como a própria idade da mulher, as limitações físicas e o cansaço, a não procura por informação, problemas de saúde, ansiedade, medo da cesárea e medo da amamentação.

*Não está sendo fácil, não, e a idade não ajuda, né? É muito cansativo. Quando a gente é mais jovem, tem mais pique, agora é muito cansativo, e ainda tenho asma pra piorar.* (G1)

*Essa gestação está bem diferente em relação ao corpo, dor. Pra andar, tem que ser mais devagar, acho que por causa da idade mesmo, me afetou bastante isso.* (G2)

*Fisicamente, meu corpo tá diferente e, como não sou mais uma menina, eu sinto o tranco, fico cansada, mesmo tendo condicionamento, porque sempre fiz exercícios.* (G6)

*Eu nem procurava me informar com nada, porque, quanto mais você se informa, mais pilhada você fica, então eu não fui atrás de nada, nenhum tipo de informação sobre gestação de risco, nada disso, não fui procurar nada, eu me abstive disso aí tudo.* (G4)

*Me preparei, na verdade, a gestação inteira pra um parto normal, e agora vem uma cesárea, entendeu, então você se preparar pra uma coisa e chegar nos 45 minutos do último tempo e ter que saber que vai fazer uma cesárea aí realmente deixa a gente meio pilhada, nervosa, mas eu fico procurando não pensar.* (G5)

*Eu tenho um pouco de medo da amamentação, na verdade. Eu assisto algumas coisas, mulheres falando que é difícil, que precisa de muita persistência, machuca, essas coisas.* (G6)

Como fatores comunitários que poderiam facilitar a transição, identificou-se o apoio da família e do companheiro, assim como da comunidade/vizinhança e do serviço de saúde.

*A família é unida. Sempre que precisa, eles ajudam, minhas irmãs, meu filho.* (G2)

*Aí, além da psicóloga, eu fui lidando com o apoio de família. Foi o único apoio que eu tive, família, de filha, que foi o que ajudou, mas não mais que isso.* (G4)

*Tô recebendo ajuda, né? Minha mãe tá ajudando, a minha mãe está aqui comigo, e tem o marido também, né, tirou férias agora pra poder ajudar e a mãe.* (G5)

*Às vezes, trava as pernas e eu não consigo andar, e preciso deitar e digo pra chamar uma ajuda, um vizinho, ou o pessoal do posto de saúde. O pessoal me passou o Whats do posto, aí eu mando mensagem falando se não estou bem, aí o médico vai lá me ver* […] *os vizinhos ficam atentos, então pra isso até posso contar com eles pra alguma urgência.* (G1)

*Eu estava com bastante medo, aí elas* [médica e enfermeira] *conversaram bastante comigo e eu estou mais tranquila.* (G2)

Alguns fatores comunitários poderiam inibir a transição, como ser mãe solo e falta de suporte familiar/companheiro.

*Aí quando ele* [o pai do bebê] *descobriu que eu estava grávida, ele foi lá em casa. A gente tentou ficar juntos, mas, na última ecografia, deu que era menina e, como ele sempre quis um menino, não sei se ele ficou triste, o que ele pensou na cabeça dele, então agora ele nem atende o telefone.* […] *quando você tem mãe, você se agarra na mãe, tua mãe é teu apoio. Mas eu fiquei sozinha, eu não tenho com quem conversar, em quem me apoiar. Às vezes, estou com dor e tenho que ficar quieta, tenho que aguentar.* (G1)

*Foi difícil, porque, assim, foi de um relacionamento que eu não sou casada com ele, né, a gente só era namorado e homem pula fora mesmo, né, isso é básico. Então, eu como já tenho 36 anos, já sei disso. Se homem casado já pula fora do barco, imagina o que não é, né?* (G3)

Além de outros fatores, as dimensões sociais podem interferir na inibição da transição. Algumas mulheres viviam em condições socioeconômicas precárias, com dificuldades financeiras. Ademais, elas frequentemente se depararam com barreiras sociais, como o constrangimento de uma gravidez em idade mais avançada e o estigma associado à ideia de ser uma “mãe mais velha”. Houve, também, uma culpabilização injusta por conceberem nesta fase da vida. Essas questões foram agravadas pelas imposições sociais e culturais em torno da maternidade, frequentemente vista como um papel socialmente esperado para as mulheres.

*Esses dias ele ligou pra ela e eu aproveitei e já disse que precisava comprar as coisas do bebê, fralda e tudo, porque não tenho nada, aí ele mandou 500 reais. Mas ele acha que esses 500 reais têm que durar a vida inteira.* (G1)

*É uma pergunta que eles fazem, mas que todo mundo faz. Nossa, como que você fica grávida aos 46 anos? Mas e agora como é que você vai levar isso?* […] *eu vou fazer 47 anos já o ano que vem, no começo do ano que vem, essa é a preocupação. A minha nora também está grávida, então às vezes bate aquela vergonha até, me sentindo velha pra estar grávida. Eu não estava esperando, né?* (G2)

Ressalta-se que condições facilitadoras de âmbito social não foram identificadas nos discursos.

### Padrões de Resposta: Processos e Resultados Como Elementos de Travessia Para a Transição à Maternidade Tardia

A sensação de conexão e interação revelou-se por meio da fé e da religiosidade, das relações com a família, com a comunidade/vizinhos, a fim de buscar apoio no processo de transição, além das relações com profissionais de saúde, demonstrando engajamento com cuidados de saúde e busca por informações para conectarse com a transição.

*Eu converso muito com Deus. Até ontem mesmo, eu estava deitada e pensando com Deus: “Como que pode um ser humano dentro da gente?”* […] *será que eu vou conseguir, será que eu vou vencer? Eu falo muito com Deus. Ele tem sido meu companheiro diário.* (G1)

*Eu fui comigo mesma tentando entender, aceitar, refletir sobre a vida, sobre as coisas que acontecem. Não procurei nenhuma ajuda psicológica, só minha família mesmo.* (G6)

*Então, minha família não é um ponto de apoio, é mais o pessoal da vizinhança que me socorre, mais do que a família, se eu precisar.* (G1)

*A médica e a enfermeira do posto que estão acompanhando a minha gravidez me orientam, conversam. Eu estava com medo, e elas me acalmaram, me sinto à vontade com elas, passa bastante segurança.* (G2)

*Eu tô me informando, nas consultas também eu tiro várias dúvidas, a médica é bem direta. A enfermeira que é um pouco mais, mais detalhista assim. Ela explica algumas coisas mais.* (G6)

A partir das conexões e interações, as mulheres desenvolveram autoconfiança e estratégias de enfrentamento para transicionar à maternidade tardia não planejada, como capacidade de lidar com os desafios da gestação tardia, mesmo as que são mãe solo. Demonstraram amadurecimento pessoal e entenderam todo o processo como um aprendizado para a vida.

*Eu já sabia de tudo que eu ia passar sozinha, tudo que eu vou passar na verdade, e o meu desespero foi esse* […], *mas hoje eu me vejo totalmente livre, sou totalmente independente* […] *acredito que eu amadureci bastante agora com essa gestação. Serviu pra eu terminar esse amadurecimento, sabe?* (G3)

*Eu só tenho essa alternativa, é ser mãe ou ser mãe, não tem uma outra, então eu tô confiante, eu tenho que estar, né? A gente erra, não tem jeito, erramos no primeiro, vamos errar no segundo, mas eu tô muito confiante* […], *pelo menos tentar fazer uma boa coisa, um bom trabalho, uma boa educação.* (G4)

*Eu ainda não sei qual exatamente, qual vai ser o aprendizado, mas, por enquanto, tá sendo isso, mesmo que a gente planeje, tenha em mente um caminho, as coisas podem mudar e a gente precisa se adaptar, se abrir pra uma nova experiência que não tava ali nos planos, mas que pode ser muito boa.* (G6)

Assim, também houve o desenvolvimento da capacidade de adaptar-se diante de novos desafios, papéis, comportamentos e necessidades impostos pela transição, traduzido em um certo domínio da situação. Isso também demonstrou a capacidade das mulheres de vivenciar, funcionar e estar bem em outros âmbitos da vida, apesar da condição de transição e todas as suas ambiguidades, em consequência de uma identidade fluida e integrativa.

*Então, eu estou bem perdida, mas estou conseguindo, devagar estou aprendendo a ser mãe, é uma novidade, estou aprendendo tudo, é como se fosse uma primeira gestação.* (G1)

*Eu falei: “Não eu posso, eu vou fazer sozinha, eu vou lutar sozinha, eu posso”* […] *a gente desenvolve forças pra enfrentar, eu acho que sempre a gente tem que desenvolver, porque senão a gente não vai para frente, né? Eu tive que desenvolver, tive que me adaptar ao que tá acontecendo comigo.* (G3)

*Melhor tentar ver as coisas como um processo que vai se construindo. A gente vai compreendendo, se adaptando e tentando fazer dar certo do jeito que cabe na nossa vida, no nosso modo de viver.* […] *tento pensar nessa gestação, assim, com os pés no chão e cabeça tranquila, se acalmar e colocar na cabeça que era pra ser assim e encarar da melhor forma.* (G6)

*Tenho minha renda, meu trabalho, estudo, eu faço o técnico de enfermagem, dou conta da minha família, assim, não dependo de ninguém, eu e meus filhos.* (G3)

## DISCUSSÃO

A transição para a maternidade tardia não planejada engloba complexidades emocionais e psicossociais. Os discursos mostram que a descoberta de uma gestação inesperada foi o ponto de entrada ou evento crítico que desencadeou a conscientização da transição para a maternidade tardia. Neste momento, as mulheres percebem a mudança significativa em suas vidas. O não planejamento dessas gestações, tanto por falha do sistema de saúde quanto por escassa adesão das mulheres e parcerias sexuais às ações de saúde, traz impactos na vida das mulheres e famílias^(11)^, e pode interferir na transição desse momento que poderia ser vivenciado de forma tranquila. A questão da idade remete a anomalias, síndromes e incapacidade de parir naturalmente. A vida cotidiana também causa preocupação, pois altera as relações, finanças e trabalho^(5,12)^.

Incertezas, angústias emocionais e conflitos interpessoais, relatados pelas participantes, são marcadores desse processo transicional, pois mudanças profundas estão movimentando as experiências de vida do indivíduo para que uma nova realidade se desenvolva^(7)^. Entende-se que as transições ocorridas na vida dessas mulheres são de diferentes tipos e padrões e estão interconectadas, como transições laborais, relacionais e etárias, que se caracterizam como situacionais, com mudança geral na vida, nas relações, bem como na carreira, e transições de desenvolvimento, pois precisam se adaptar a novos papéis e reconfigurar suas identidades, assim como transições de saúde/doença, quando relatam impacto físico, desafios de saúde e preocupações emocionais.

Assim, as transições também ocorreram em múltiplos padrões, sequenciais e simultâneos, influenciando a experiência de cada mulher. A maioria das gestantes enfrenta transições múltiplas, pois não só está se adaptando à ideia de ser mãe novamente, muitas vezes após um longo intervalo, mas também está lidando com mudanças situacionais, de desenvolvimento e de saúde/doença. Dessa forma, as experiências de transição das gestantes estão claramente interligadas e partem da descoberta da gravidez, que as leva a ajustes pessoais e emocionais, seguidas por adaptações físicas e sociais. Essa progressão reflete a transição sequencial e simultânea das mulheres através de várias etapas de adaptação. Desse modo, a enfermagem tem papel importante de apoio nesse período de movimentos, ao compartilhar informações e prestar cuidados de enfermagem específicos que estejam vinculados às necessidades individuais de cada mulher.

A transição para a maternidade em gestações em idade avançada, sobretudo as não planejadas, pode ser facilitada por condições pessoais. A fé e a religiosidade se mostraram uma dimensão espiritual que pode facilitar processos, resultando em efeitos psicológicos favoráveis, graças à crença, o que, por sua vez, ajuda a elevar a autoconfiança e a aceitação do desafio imposto e, assim, têm um impacto positivo no processo transicional, especialmente na saúde mental^(12)^.

Contudo, fatores pessoais podem também dificultar essa transição, como exemplificado nos discursos, como a pressão da idade, limitações físicas atreladas a essa idade, cansaço, doenças pré-existentes. Algumas mulheres que engravidam em idade avançada podem experimentar esse tipo de situação, especialmente se não planejaram a gravidez. Elas podem se questionar sobre a falha na contracepção, que é uma responsabilidade socialmente atribuída à mulher, e que, ao engravidar na idade “errada”, colocam em risco a saúde do bebê^(5,12)^.

Além disso, quando se fala em gestação tardia, as mulheres podem se sentir envergonhadas e estigmatizadas por serem mães “velhas”^(6)^. O Ministério da Saúde recomenda que a idade “ideal” para engravidar é entre 20 e 34 anos, pois, após isso, há risco de intercorrências materno-fetais^(13)^. Porém, a idade por si só pode não constituir fator de risco se a mulher for saudável e houver um bom controle de pré-natal^(5)^. Ademais, tem-se uma construção sociocultural de que mulheres mais velhas ou próximas ao período menopáusico não têm vida sexual ou que são incapazes de engravidar devido à diminuição da fertilidade^(8)^.

A gestação tardia, apesar de ser uma tendência, ainda causa espanto, e mulheres nessa situação são alvo de comentários insensíveis e preconceituosos^(6,8)^. Tais situações podem resultar em desconexão com a maternidade e isolamento, impedindo o desenvolvimento de habilidades adaptativas, indicativo de uma transição desafiadora.

As condições comunitárias, como o apoio social familiar e do(a) parceiro(a), têm papel essencial como agentes socializadores na vida das pessoas, transmitindo não apenas apoio e orientação, mas também valores, crenças e tradições. Essa interrelação e interação com micro e macrossistemas auxilia ou dificulta o processo de transição, assim como outros contextos sociais em que se incluem as redes ampliadas, como vizinhos, amigos e serviços de saúde^(12,14,15)^.

No Brasil, há uma considerável parcela de mulheres que são mães, porém não estão envolvidas em um casamento formal ou relações conjugais. Isso pode ocorrer tanto devido a escolhas intencionais quanto por razões socioculturais, como abandono ou falta de envolvimento do pai na criação dos filhos. A maternidade solo é um problema social e uma realidade significativa no Brasil, em que elas frequentemente enfrentam uma série de experiências desiguais e discriminatórias que as afetam. Isso inclui o estigma associado à falta de um parceiro, juntamente com outros fatores que têm um impacto considerável em suas vidas cotidianas, como obstáculos para acessar determinadas instâncias sociais, uma carga de trabalho mais pesada e disparidades salariais^(16)^.

Todos esses fatores, atrelados ainda ao estigma da idade e à falta de outros tipos de apoios sociais, podem desencadear indicadores de processo e de resultados que conduzem as mulheres a vulnerabilidades, principalmente psicossociais, impedindo-as de desenvolver autoconfiança, estratégias de enfrentamento, domínio situacional. Esses indicadores de processo e resultado são essenciais para que se avalie a capacidade das mulheres de gerenciar as mudanças advindas da transição, que requerem novas habilidades, sentimentos, objetivos, comportamentos ou funções^(7)^.

Nesse sentido, ficou evidente que a gravidez inesperada desencadeou uma revisão profunda de sua autoimagem e papéis sociais. Esse fluxo identitário, que tem por base a transformação e a autopercepção, é observado nos discursos, em que elas articulam sentimentos de incerteza e redefinição. A transição não é apenas física, mas profundamente enraizada nas dimensões psicológica e social, influenciada tanto por fatores internos quanto pelo apoio (ou sua ausência) do entorno social. As entrevistadas refletem sobre como a maternidade tardia alterou suas prioridades, perspectivas de vida e relações interpessoais, ilustrando como a identidade feminina, particularmente em contextos de maternidade não planejada, está em constante evolução e renegociação.

Nos últimos anos, tem-se observado uma mudança significativa na percepção da gestação em idade avançada e no papel das mulheres na sociedade. Historicamente, a expectativa era que as mulheres tivessem filhos em idades mais jovens, mas o aumento da idade média para a primeira gravidez reflete mudanças nas prioridades e nas oportunidades educacionais e profissionais disponíveis para as mulheres. Essas mudanças também implicam desafios únicos para a saúde mental, como o estresse associado ao equilíbrio entre carreira e maternidade, e o impacto do estigma social em mulheres que optam por ter filhos em idades mais avançadas^(9)^.

A literatura indica que mulheres em gestação tardia podem enfrentar níveis elevados de ansiedade e estresse, muitas vezes exacerbados pela percepção de que estão “fora do tempo” para a maternidade. Além disso, essas mulheres podem sentir uma pressão adicional para atender às expectativas sociais de serem mães e profissionais bem-sucedidas, o que pode impactar negativamente sua saúde mental^(14)^. É crucial, portanto, que haja um suporte psicológico e social adequado, incluindo a implementação de políticas públicas que reconheçam e abordem essas necessidades específicas. Programas de apoio psicológico, grupos de suporte e orientação especializada podem ser ferramentas importantes para ajudar essas mulheres a transpassar pelos desafios emocionais e sociais da gestação tardia.

Pesquisas recentes têm destacado a importância de reconhecer e abordar os aspectos psicossociais e emocionais da maternidade em idade avançada. Estudos sugerem que intervenções direcionadas podem não apenas melhorar a saúde mental das gestantes, mas também promover uma experiência de maternidade mais positiva e satisfatória^(12,14)^.

É fundamental que os profissionais de saúde, em especial os enfermeiros, estejam cientes dessas complexidades da transição para a maternidade tardia não planejada e ofereçam terapêuticas de enfermagem a fim de facilitar e inspirar processos saudáveis e respostas positivas de transição. Isso inclui intervenções de enfermagem pautadas em educação em saúde, informações e orientações precisas, capacitação da mulher para enfretamentos, estabelecimento de metas, apoio emocional e, quando necessário, encaminhamento para serviços de apoio psicológico ou assistência social. Para isto ocorrer, é necessário que haja interação e diálogo entre enfermeiro e mulher/família, a fim de identificar marcos e pontos críticos, redes de apoio e demais necessidades individualizadas.

O estudo contribui para o avanço do conhecimento em enfermagem na medida em que evidencia a complexidade psicossocial e emocional da maternidade tardia não planejada, destacando as múltiplas dimensões que afetam as mulheres nessa transição. Diante disso, é imprescindível que a enfermagem amplie seu olhar para além dos aspectos fisiológicos e patológicos da gravidez, abordando as dimensões subjetivas, comportamentais e interpessoais. A identificação de áreas específicas para apoio e intervenções de enfermagem orientadas pela perspectiva do cuidado de si, qualidade de vida, bem-estar e desenvolvimento de habilidades é crucial para preparar a mulher para questões que transcendem as preocupações biomédicas.

Além disso, refletindo sobre a atual política de planejamento reprodutivo no Brasil, observa-se a necessidade de uma abordagem mais integrada e desfragmentada. A disponibilização de contraceptivos, embora seja importante, é apenas uma faceta de uma política pública mais abrangente que deve incluir ações educativas, orientativas e clínicas, formando um tripé indivisível para a proteção da saúde sexual e reprodutiva. Portanto, urge o fortalecimento das políticas públicas nesse campo, com incentivos governamentais e modernização das estratégias de operacionalização pela Rede de Atenção à Saúde brasileira.

Como a transição se dá ao longo do tempo, compreende-se que uma limitação pode ter sido a coleta apenas no período de gravidez, prejudicando a percepção da vivência dessas mulheres após o parto, quando, inferencialmente, pode ser finalizada a transição. Além disso, dados de apenas uma região e em pequena quantidade podem limitar a generalização. Porém, entende-se que os achados aqui apresentados não podem ser desprestigiados, pois apresentam vivências importantes sobre o período gestacional de uma população pouco estudada no Brasil.

Ainda, ressalta-se como uma limitação a dificuldade de aceite das gestantes para a efetivação das entrevistas, mesmo que a opção via telefone fosse ofertada, a fim de viabilizar flexibilidade de tempo de cada mulher.

## CONCLUSÃO

Este estudo aprofunda a compreensão dos desafios enfrentados pelas mulheres que passam por uma transição para a maternidade em idade avançada não planejada, ao apresentar *insights* sobre a experiência dessas mulheres, destacando a complexidade do processo de adaptação, a partir de uma variedade de fatores que podem facilitar ou inibir a transição bemsucedida para a maternidade tardia. A partir disso, é possível refletir sobre intervenções individualizadas destinadas a apoiar as mulheres por meio de orientações específicas sobre cuidados pré-natais, planejamento familiar e estratégias de enfrentamento.

Futuras pesquisas podem explorar ainda mais as nuances dessa experiência, visando aprimorar as práticas de cuidado e contribuir para o desenvolvimento de políticas públicas mais eficazes. A enfermagem brasileira pode avançar no desenvolvimento de capacitações especializadas, desenvolvimento de protocolos de atendimento que considerem particularidades da gravidez tardia e fomentar pesquisas contínuas na área sobre melhores práticas. Por fim, este estudo ressalta a importância de se abordar a maternidade tardia não planejada com sensibilidade e compreensão, considerando as necessidades únicas dessas mulheres e suas famílias, em busca de um processo de transição mais saudável e empoderador.
